# The Rebirth of Waste Cooking Oil to Novel Bio-based Surfactants

**DOI:** 10.1038/srep09971

**Published:** 2015-05-06

**Authors:** Qi-Qi Zhang, Bang-Xin Cai, Wen-Jie Xu, Hong-Ze Gang, Jin-Feng Liu, Shi-Zhong Yang, Bo-Zhong Mu

**Affiliations:** 1 State Key Laboratory of Bioreactor Engineering and Institute of Applied Chemistry, East China University of Science and Technology, Shanghai 200237, P.R. China; 2 Shanghai Collaborative Innovation Center for Biomanufacturing Technology, Shanghai 200237, P.R. China

## Abstract

Waste cooking oil (WCO) is a kind of non-edible oil with enormous quantities and its unreasonable dispose may generate negative impact on human life and environment. However, WCO is certainly a renewable feedstock of bio-based materials. To get the rebirth of WCO, we have established a facile and high-yield method to convert WCO to bio-based zwitterionic surfactants with excellent surface and interfacial properties. The interfacial tension between crude oil and water could reach ultra-low value as 0.0016 mN m^−1^ at a low dosage as 0.100 g L^−1^ of this bio-based surfactant without the aid of extra alkali, which shows a strong interfacial activity and the great potential application in many industrial fields, in particular, the application in enhanced oil recovery in oilfields in place of petroleum-based surfactants.

Most of the surfactants used in industrial fields are petroleum-based. Bio-based surfactants have attracted much attention from scientific and industrial fields due to their renewable feedstock and environmentally friendly application^1^,^2^,^3^,^4^,^5^,^6^. WCO is a renewable feedstock with enormous quantities and it could not be used in foods anymore. In 2013, the consumption of vegetable oils and animal fats for foods in China was about 30 million tons, which generated 15% (about 4.5 million tons) waste cooking oils^7^. Seeking an effective and safe process to process such vast WCO is one of the most decisive issues for current society and converting WCO to diverse types of functional materials has become a major focus of research interest. Recent examples of its successful exploitation in non-food applications include as a feedstock in the manufacture of biodiesel^8^ and aviation fuel^9^, which have mitigated the energy shortages. If taken as the starting material for the production of surfactants, WCO will have a more broad application as substitutes for entirely petroleum-based surfactants. The major components of WCO are triglycerides esters of glycerol with three long-chain fatty acids (>C12), given priority to oleic acid and linoleic acid. These abundant fatty acids are advantageous choices for the starting materials of bio-based surfactants. In recent years, some of the bio-based surfactants derived from fatty acids have been documented^2^,^3^,^4^,^5^,^6^. The share of entirely petroleum-based surfactants in surfactant production is declining gradually and biomass is seen as one of the best substitutes to replace fossil resources^10^,^11^,^12^,^13^,^14^,^15^,^16^,^17^. There are also researches about surfactants derived from vegetable oils, by commonly focusing on anionic surfactants^18^,^19^. The calcium tolerance of anionic surfactant is inferior to zwitterionic surfactants. Zwitterionic surfactants, which are electrically neutral compounds, have small effective headgroup area thanks to the proximity of their positive and negative charges^3^. The synthesis and interfacial properties of zwitterionic surfactants applied in enhanced oil recovery have been reported^20^,^21^. But the research about bio-based zwitterionic surfactants derived from WCO and their application on enhanced oil recovery is still an uncultivated land. Here we report a facile and high-yield approach to get WCO’s rebirth by converting it to a novel bio-based zwitterionic surfactant with outstanding surface and interfacial properties.

## Results

### Synthesis, yield and characterization of bio-based zwitterionic surfactants from WCO

**Figure 1:**
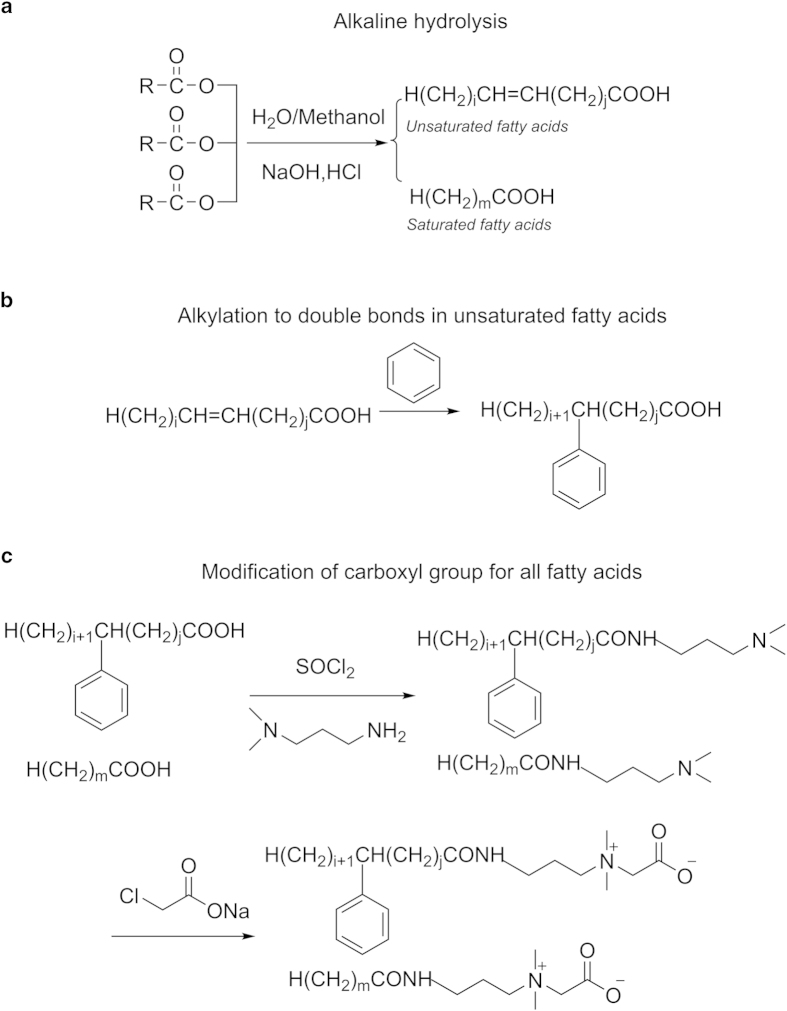
The synthetic method to convert WCO to bio-based zwitterionic surfactants.

The collected WCO was pretreated through filtration and centrifugation. The oil phase was acidified, filtered, washed with water and then dried. In the first step (as shown in Fig. 1), the mixture of fatty acids from WCO was prepared using alkaline hydrolysis in yield of 98.0%. The methyl trans-esterification products of WCO were analyzed using GC-MS chromatography. The main fatty acid composition of WCO was presented in Table 1. The composition of WCO would change in different area or different diet custom. Nonetheless, the modification of fatty acids mixtures according to our previous work is applicative^21^. The chemical modification of instable double bonds in oleic/linoleic/linolenic acid molecules was first carried out through Friedel–Crafts alkylation reaction using benzene as alkylation reagent and AlCl_3_ as catalyst. Then the chemical modification of the carboxyl group of all fatty acids was accomplished by acyl chlorination with sulfoxide chloride, amidation with *N*, *N*-dimethyl-1,3- propylenediamine and quaternization with sodium chloroacetate to obtain bio-based zwitterionic surfactants, *N*-phenyl fatty amidopropyl-*N*,*N*-dimethylcarboxylbetaine (PFAPMB), yielded 84.9%. ESI HRMS (m/z): [M+Na]^+^ calcd for: C_23_H_46_N_2_O_3_ (C16:0), 421.3406, C_25_H_50_N_2_O_3_ (C18:0), 449.3719, C_31_H_50_N_2_O_3_ (C18:2:pheynl), 521.3719, C_31_H_52_N_2_O_3_ (C18:1:phenyl), 523.3875, C_31_H_54_N_2_O_3_ (C18:0:pheyl), 525.4134; found: 421.3416, 449.3722, 521.3674, 523.3884, 525.4045; C_23_H_46_N_2_O_3_ (C16:0) : C_25_H_50_N_2_O_3_ (C18:0) : C_31_H_50_N_2_O_3_ (C18:2:pheynl) : C_31_H_52_N_2_O_3_ (C18:1:phenyl) : C_31_H_54_N_2_O_3_ (C18:0:pheyl) = 0.24:0.17:0.11:0.80:1.0. ^1^H NMR (400 MHz, CDCl_3_): δ 7.159-7.116 (-C_6_**H**_**5**_), 6.921 (-CO-N**H**-), 4.144-4.090 (-C**H**=C**H**-), 3.852-3.847 (-NH-C**H**_**2**_-CH_2_-), 3.672-3.645 (-N(CH_3_)_2_-C**H**_**2**_-COO), 3.483 (-C**H**_**2**_-N(CH_3_)_2_-), 3.221 (-N(C**H**_**3**_)_2_-), 2.447-2.428 (-C**H**-C_6_H_5_), 2.142 (-C**H**_**2**_-CO-), 1.968-1.959 (-NH-CH_2_-C**H**_**2**_-CH_2_-N(CH_3_)_2_-), 1.578-1.563 (-C**H**_**2**_-CH-; -C**H**_**2**_-CH_2_-CO-), 1.255-1.238 (-(C**H**_**2**_)_n_-CH_3_), 0.890-0.856 (-CH_2_-C**H**_**3**_).

### Surface properties

**Figure 2:**
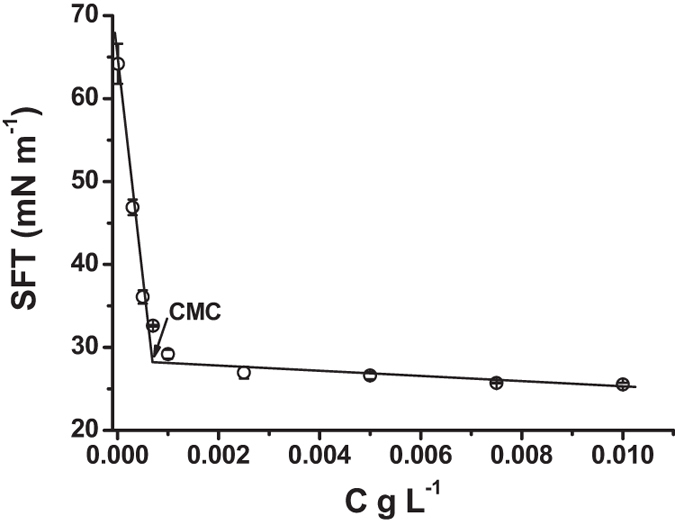
Variation of the surface tensions with the concentration of PFAPMB at 25.0 degree C. The error bars represent standard deviations of the mean of triplicate measurements.

Variation of the surface tensions (SFT) with the concentration of PFAPMB at 25.0 ^o^C was illustrated in Fig. 2. Surface properties (calculation method and formula were listed in Supplementary Information, Section “Surface properties”) of PFAPMB were listed in Table 2. As seen in Fig. 2, SFTs were decreased gradually with the increase of surfactant concentrations. An obvious inflection point was obtained in the plot of SFT versus surfactant concentration, as indicated e.g. by the arrow in Fig. 2, which is named as the critical micelle concentration (CMC). The CMC of PFAPMB was extremely low as 0.734 mg L^−1^ with the SFT at CMC (SFT_CMC_) of 28.4 mN m^−1^. The CMC of PFAPMB was three orders of magnitude lower than other bio-based surfactants and the max surface excess concentration was almost two times of others’^18^.

### Interfacial properties

**Figure 3:**
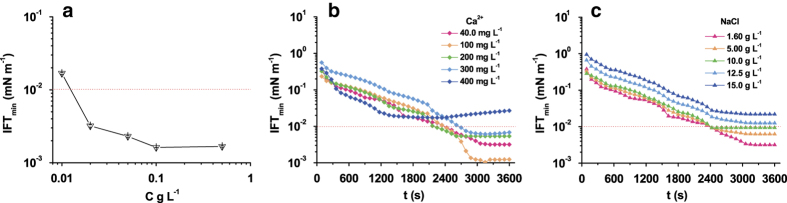
The interfacial properties of PFAPMB. (**a**) The minimum of interfacial tensions between Daqing crude oil and different concentration PFAPMB solutions at 50.0 degree C, The error bars represent standard deviations of the mean of triplicate measurements; (**b**) Dynamic interfacial tensions between Daqing crude oil and 0.020 g/L PFAPMB solutions with adding extra Ca^2+^ at 50.0 degree C; (**c**) Dynamic interfacial tensions between Daqing crude oil and 0.020 g/L PFAPMB solutions with adding extra NaCl at 50.0 degree C.

Dynamic interfacial tensions (IFT) between Daqing crude oil (the composition and fundamental properties of the crude oil were listed in Supplementary Information, section “Material”) and water at the different concentrations of PFAPMB in water solutions at 50.0 ^o^C (the average temperature of the stratum in Daqing oil field, China) were shown in Fig. 3(a).

When the interfacial tension between the surfactant solution and crude oil arrives at ultra-low level (<10^−2 ^mN m^−1^), the efficiency of oil recovery will be remarkably enhanced^22^,^23^. As Fig. 3(a) showed, the minimum interfacial tension between oil and different concentration solutions of PFAPMB decreased gradually with the increase of concentrations. The minimum of (IFT_min_) could reach 0.0016 mN m^−1^ at the concentration of 0.100 g L^−1^ PFAPMB. Considered in the application, achieving ultra-low interfacial tension is the primary goal, the lower usage of the surfactants is also preferred. So, the subsequent experiment selected the concentration of 0.020 g L^−1^ as the research basis, at which concentration the IFT_min_ was 0.0032 mN m^−1^. There is an interesting phenomenon, when the concentration of PFAPMB was much lower than single surfactant in our precious work^21^, better reduction of IFT could be obtained. This is due to the synergism among the surfactant analogues. Mixture-chain surfactants are known to have superior surface/interfacial performance to single-chain counterparts owing to the enhanced ability of molecular self-assembly^18^,^19^. In the reaches of surface chemistry, several surfactants were frequently compounded together^24^,^25^, in order to reduce the dosage of surfactants and to achieve lower surface/interfacial tensions. In this case, the starting material was the mixture of fatty acids; the final product was also a mixture, and PFAPMB is a naturally surfactant compound system showing great synergism.

The effect of adding extra Ca^2+^/NaCl on dynamic interfacial tensions between Daqing crude oil and 0.020 g L^−1^ PFAPMB solutions at 50.0 ^o^C were tested respectively and the dynamic interfacial tensions were shown in Fig. 3(b) and (c). The influence of electrolyte on interfacial properties of PFAPMB were presented in Table 3. From the results of Fig. 3(b), 3(c) and Table 3, PFAPMB has strong electrolyte tolerance. When the concentration of Ca^2+^ was below 300 mg L^−1^, the dynamic interfacial tensions between Daqing crude oil and 0.020 g L^−1^ PFAPMB solutions could remain in ultralow range. When the concentration of NaCl was below 10.0 g L^−1^, *viz.* the total dissolved substance was below 15000 mg L^−1^, PFAPMB solutions still showed excellent interfacial properties. Salinity and divalent ions have important effect on the interfacial properties of surfactants. The application of anion surfactants has been limited due to the complexation between surfactants and ions in high-salinity and high-calcium oil fields. In China, the salinities of most oil fields are commonly below 16000 mg L^−1^ and the concentrations of Ca^2+^ are below 300 mg L^−1^ 26. The present results suggested that PFAPMB had strong electrolyte tolerance, and it could remain good interfacial properties in a wide range of salinity. This observation had provided the possibility for its application in most oil fields of China.

### Wetting, emulsification and foaming properties

The contact angle *θ*_*average*_ of 0.500 g L^−1^ PFAPMB solution was 38.79 ^o^, which was measured after adding the solution for 60 s. The emulsification property was determined by recording the time taken for separation aqueous phase solutions from adequately blending mixture of liquid paraffin and 0.500 g L^−1^ PFAPMB solution. The time for separation 3 mL and 5 mL aqueous phase solutions were 170 s and 302 s, respectively. The foaming property of 0.500 g L^−1^ PFAPMB solution was obtained according to Ross-Miles test at 40 ^o^C. The initial foam height and foam height after 10 min were 73 mm and 70 mm, respectively.

### Biodegradation estimation

Ultimate biodegradation scores for bio-based zwitterionic surfactants in PFAPMB were from 2.64 to 2.92. The bigger score indicates that the expected total degradation speed is faster. Scores above 2.8 are in the order of “weeks”, and scores between 2.0 to 2.8 were in order of “months”. The expected total degradation time was in the order of “months” for PFAPMB. These results also demonstrate that PFAPMB are biodegradable, and the degradation time is suitable for EOR, in which the stability of surfactants for several months is needed.

## Discussion

In this work, the rebirth of WCO by converting it to bio-based zwitterionic surfactants was achieved via a facile and high-yield chemical modification. It is satisfactory that PFAPMB exhibits excellent interfacial properties, and the interfacial tensions between Daqing crude oil and 0.100 g L^−1^ PFAPMB solutions could reach ultralow value, as 0.0016 mN m^ 1^. Ultra-low IFT between oil and water is necessary for EOR in oilfields in the absence of alkali. As a comparison, alkali, was added with petroleum-based surfactants to reduce oil-water interfacial tension to ultralow values and enhance oil recovery currently^27^,^28^,^29^, which has resulted in well bore scaling, stratum damage and permeability decline^30^,^31^. Thus the bio-based zwitterionic surfactants derived from WCO could be considered as a potent alternative in oil recovery to increase oil displacement efficiency and to reduce the dosage of alkali in oil fields. Additionally, the solution of PFAPMB showed good spreadability on the hydrophobic solid substrates, stable emulsification property, strong foaming power and appropriate biodegradability. These results all demonstrate that PFAPMB has wide potential applications.

In view of abundant starting material, outstanding surface/interfacial properties and biodegradability, bio-based zwitterionic surfactants derived from WCO will be of great interests for many surfactants application fields, such as detergents, cosmetics, bioremediation, etc, and more specifically in enhanced oil recovery. At the same time, the negative impact of WCO to environment and human could be considerably reduced. Waste cooking oil is not waste anymore, it could get its gorgeous rebirth and create more values.

## Methods

### Pretreatment of WCO

WCO collected from canteens and restaurants was firstly pretreated through filtration and centrifugation. The upper oil phase was acidified, filtered and washed with water. The starting material was obtained after dehydration.

### Synthesis of bio-based zwitterionic surfactants derived from WCO

20.00 g WCO (saponification value: 177.83 mg KOH g^−1^) and 30 mL NaOH methanol/water solution (3 M, *v*_methanol_*/v*_water _= 2:1) were added into a round bottom flask. The reaction mixture was refluxed at 70 °C for 6 h. After the hydrolysis, the mixture was neutralized and further acidized using hydrochloric acid (6 M), until the pH value of the solution was about 2. Then 50 mL hot water was added to separate fatty acids and glycerol. The aqueous phase was removed. The oil phase was washed by hot water for 5 times, and the pH value of the aqueous phase was about 6. Then the oils phase was dried using cyclohexane as water-carrying agent. The acid values before and after the hydrolysis process were 1.37 mg KOH g^−1^ and 174.33 mg KOH g^−1^. So the hydrolysis efficiency was 98.0%. 5.00 g (18 mmol) of mix fatty acids and 2.40 g (18 mmol) of AlCl_3_ were added to 25 mL benzene in a round-bottom flask. The reaction mixture was refluxed at 65 °C, and anhydrous CaCl_2_ was placed in a drying tube above the reactor. After 6 h of reaction, the product was washed by 15 mL hydrochloric acids (1 M) for 3 times, then excess benzene was removed on a rotary evaporator giving Phenyl fatty acids (PFA), 5.90 g, yielded 98.0%. A solution of PFA (5.90 g, 18 mmol) in trichloromethane (25 mL) was added dropwise to a drying flask with 1.45 mL SOCl_2_ (20 mmol) in it, and the mixture was stirred at 40 °C for 2 h. Then the mixture was distilled to remove the solvent and excess SOCl_2_. The residue was dissolved in 10 mL acetone, and 2.30 mL *N*,*N*-dimethyl-1,3-propanediamine (20 mmol) was slowly added in it at 0 °C. The reaction mixture was then warmed to 40 °C and stirred for 2 h. The excess diamine and solvent were removed on a rotary evaporator giving *N*-phenyl fatty amidopropyl–*N*,*N*-dimethylamine (PFAPMA), 6.76 g, yielded 91.5%. PFAPMA were quaternized using sodium chloroacetate with a molar ratio of 1:1.25 in a solvent mixture of methanol and water (*v*_methanol_*/v*_water_ = 1:4). The final products were refluxed at 75 °C for about 12 h; the mixture was vaporized off under reduced pressure, then dissolved in ethanol and filtered. The filter liquor was distilled to remove ethanol and purified by recrystallization in ethyl acetate. 7.29 g precipitate, *N*-phenyl fatty amidopropyl-*N*,*N*-dimethylcarboxylbetaine (PFAPMB), was received and yielded 84.9%. ESI HRMS: m/z [M+Na]^+^ calcd for: 421.3406 (C16:0), 449.3719 (C18:0), 521.3719 (C18:2:pheynl), 523.3875 (C18:1:phenyl), 525.4134 (C18:0:pheyl); found: 421.3416, 449.3722, 521.3674, 523.3884, 525.4045. ^1^H NMR (400 MHz, CDCl_3_): δ 7.159-7.116 (-C_6_**H**_**5**_), 6.921 (-CO-N**H**-), 4.144-4.090 (-C**H**=C**H**-), 3.852-3.847 (-NH-C**H**_**2**_-CH_2_-), 3.672-3.645 (-N(CH_3_)_2_-C**H**_**2**_-COO), 3.483 (-C**H**_**2**_-N(CH_3_)_2_-), 3.221 (-N(C**H**_**3**_)_2_-), 2.447-2.428 (-C**H**-C_6_H_5_), 2.142 (-C**H**_**2**_-CO-), 1.968-1.959 (-NH-CH_2_-C**H**_**2**_-CH_2_-N(CH_3_)_2_-), 1.578-1.563 (-C**H**_**2**_-CH-; -C**H**_**2**_-CH_2_-CO-), 1.255-1.238 (-(C**H**_**2**_)_n_-CH_3_), 0.890-0.856 (-CH_2_-C**H**_**3**_).

### Characterization

GC-MS spectra were recorded on an Agilent 6890N network GC system and 5975 inert Mass Selective Detector. ESI HRMS spectra were recorded on the Waters LCT Premier XE Mass Spectrometers. ^1^H NMR spectra of all the intermediates and final surfactants were recorded on a Bruker Avance 400 spectrometer (400 MHz) in CDCl_3_ at room temperature.

### Surface tension measurement

The surface tensions of the aqueous solution at different surfactant concentrations were measured by using a DCAT 21 tensiometer (Dataphysics, Germany) with the plate method and the temperature was controlled at 25.0 ± 0.1 °C. Before each measurement, the quartz plate was briefly heated above an alcohol burner until glowing. In order to attain equilibrium conditions, the quartz plate should be cooled to room temperature and dipped in the solution for a while. The measurement should be repeated three times and an average value was obtained. The surface tension between air and double distilled water was 71.8 mN m^−1^ at 25 °C.

### Interfacial tension measurement

Interfacial tensions between Daqing crude oil and water were measured by the spinning-drop method at 50 ± 0.1 °C using SVT 20 tensiometer (Dataphysics, Germany). Equilibrium was affirmed to be obtained when successive values taken at 5 min intervals agreed to within 0.001 mN m^−1^. The volumetric ratio of water to oil in the spinning-drop tensiometer is about 2000. Daqing crude oil was used as oil phase. Surfactant solutions were prepared with Daqing oil field simulated formation water and the concentration of the surfactants was 0.020 g L^−1^. The formation water consists of 112.7 mg L^−1^ CaCl_2_, 42.8 mg L^−1^ MgCl_2_, 1597.1 mg L^−1^ NaCl, 17.0 mg L^−1^ Na_2_SO_4_, 381.6 mg L^−1^ Na_2_CO_3_ and 3176.0 mg L^−1^ NaHCO_3_. The total dissolved substance is 5327.2 mg L^−1^. The interfacial tension between Daqing crude oil and simulated formation water was 9.70 mN m^−1^ at 50 °C.

### Contact angle measurement

The contact angles were measured by using the sessile drop technique. The apparatus consisted of a camera with a micro lens and a horizontal platform. The platform was connected to the thermostat to control temperature. Before measurement, the platform environment was preheated at 25 ± 1 °C by switching on the thermostat. When the temperature reached 25 °C, the solid substrate was placed in middle of the platform. A 2 μL solution drop was introduced onto the solid substrate through a microsyringe. The contact angles were measured by taking photographic images after adding the solution for 60 s. The contact angles were determined directly from the photographs by drawing tangent lines between the liquid drop and the solid surface. The measurements were repeated 5 times for every samples and the average angle value was figured out. The average contact angle on the three phase contact gas/double distilled water/solid was 92.04 ^o^ at 25 °C.

### Emulsification property measurement

The emulsification properties of bio-based zwitterionic surfactants were obtained at room temperature. It was determined by giving vigorous downward stokes to a mixture of 10 mL 0.500 g L^−1^ bio-based zwitterionic surfactants solution and 10 mL liquid paraffin in a graduated test tube. The time taken for separation of 3 mL and 5 mL aqueous phase solution was recorded (t/s). The recorded time was repeated for 3  imes.

### Foaming property measurement

The foaming property of bio-based zwitterionic surfactants was obtained according to the Ross-Miles test at 40 ^o^C^19^. The foam heights at 0 min and 10 min were recorded (h/mm). The recorded height was repeated for 3 times.

### Ultimate biodegradation estimation

Ultimate biodegradation estimation of PFAPMB was determined by EPI Suite, BIOWIN3 biodegradation model, which is frequently used to estimate the degradation of organic chemicals^32^.

### Chemicals

AlCl_3_ (99%, Aladdin, Shanghai), *N*, *N*-dimethyl-1, 3-propanediamine (99%, GC, Sigma, Shanghai), Sodium chloroacetate (99%, Aladdin, Shanghai) were used without further purification. All other chemicals used were of analytical reagent grade. WCO was collected at canteens and restaurants. The contents of fatty acids of WCO were analyzed by GC-MS.

## Author Contributions

This study was designed by Q.-Q.Z., H.-Z.G., J.-F.L., S.-Z.Y. and B.-Z.M. Q.-Q.Z. performed the chemical synthesis of bio-based zwitterionic surfactants derived from WCO, measured the surface properties of the surfactants and analyzed the experimental results. B.-X.C. performed the interfacial tension measurements. W.-J.X. performed the hydrolysis of WCO. Q.-Q.Z. wrote the manuscript, assisted by all co-authors. All authors reviewed the final manuscript.

## Additional Information

**How to cite this article**: Zhang, Q.-Q. *et al.* The Rebirth of Waste Cooking Oil to Novel Bio-based Surfactants. *Sci. Rep.* **5**, 09971; doi: 10.1038/srep09971 (2015).

## Supplementary Material

Supplementary Information

## Figures and Tables

**Table 1:** The contents of main fatty acids in WCO.

	**Palmitic Acid C16:0**	**Stearic Acid C18:0**	**Oleic Acid C18:1**	**Linoleic Acid C18:2**	**Linolenic Acid C18:3**
wt %	18.16	8.12	40.72	25.81	7.19

**Table 2:** Surface properties of PFAPMB.

**The resource of surfactants**	**CMC (mg L**^**−1**^)	**SFT**_**CMC**_ **(mN m**^**−1**^)	**SFT**_**min**_ **(mN m**^**−1**^)	**Γ**_**max**_ **(μmol m**^**−2**^)	**A**_**min**_ **(nm**^**2**^**/molecule)**
Waste Cooking Oil	0.734	28.4	25.5	2.66	0.62

**Table 3:** Interface properties of PFAPMB on electrolyte tolerance.

	**Ca**^**2+**^ **concentration**					**NaCl concentration**				
	**40 mg L**^**−1**^	**100 mg L**^**−1**^	**200 mg L**^**−1**^	**300 mg L**^**−1**^	**400 mg L**^**−1**^	**1.60 g L**^**−1**^	**5.00 g L**^**−1**^	**10.0 g L**^**−1**^	**12.5 g L**^**−1**^	**15.0 g L**^**−1**^
IFT_min_ mN m^–1^	0.0032	0.0007	0.0054	0.0068	0.0171	0.0032	0.0063	0.0094	0.0125	0.0219
IFT_equ_ mN m^–1^	0.0032	0.0014	0.0054	0.0068	0.0269	0.0032	0.0063	0.0094	0.0125	0.0219
