# Supplementary material for: The Rebirth of Waste Cooking Oil to Novel Bio-based Surfactants
Source: Sci Rep. 2015 May 6;5:9971. doi: 10.1038/srep09971 (PMC4421825; doi:10.1038/srep09971)
Supplement: Supplementary Information [file srep09971-s1.doc]

**Supplementary Information**

**The Rebirth of Waste Cooking Oil to Novel Bio-based Surfactants**

Qi-Qi Zhang^1^, Bang-Xin Cai^1^, Wen-Jie Xu^1^, Hong-Ze Gang^1^, Jin-Feng Liu^1^, Shi-Zhong Yang^1^, Bo-Zhong Mu^1,2,*^

^1^State Key Laboratory of Bioreactor Engineering and Institute of Applied Chemistry, East China University of Science and Technology, Shanghai 200237, P.R. China

^2^Shanghai Collaborative Innovation Center for Biomanufacturing Technology, Shanghai 200237, P.R. China

^*^Corresponding Author: Bo-Zhong Mu

E-mail: bzmu@ecust.edu.cn

Phone: +86 21 64252063; Fax: +86 21 64252485

**1 Materials**

Daqing Crude Oil were dehydrated and degassed. The acid value is 0.06 mg KOH/g. The density of the crude oil is 0.84 g/cm^3^ and the viscosity was 19.2 mPa·s at 50 ^o^C. The main compositions are asphaltene (8.12 wt %), resins (24.12 wt %), hydrocarbon (65.88 wt %) and organic acids (0.13 wt %). The main hydrocarbons are alkanes from C13-C31, among which C23 was the most abundant.

The contents of main fatty acids in waste cooking oil (WCO) were analyzed from the methyl trans-esterification products of WCO using GC-MS chromatography. Total ion chromatograph of fatty acids methyl ester in WCO was showed in Figure S1.


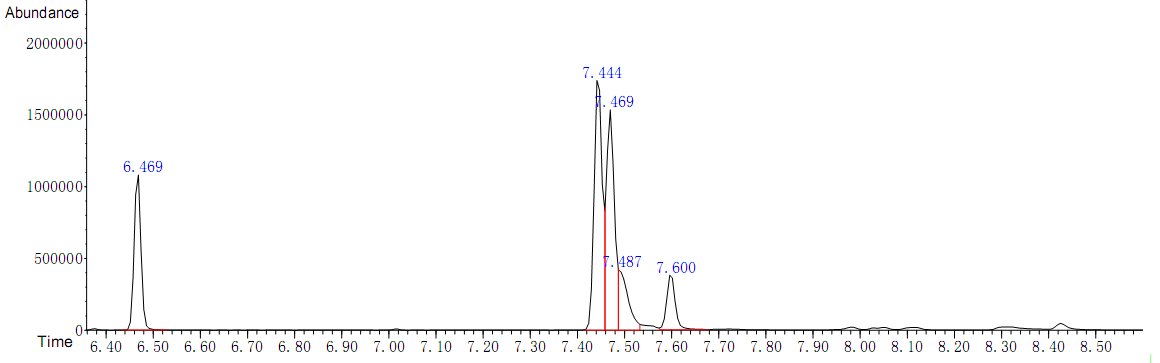


**Figure S1. Total ion chromatograph of main fatty acids methyl ester in WCO.** A 0.2 μL sample was injected in split mode (split/column flow ratio 50:1). The carrier gas was helium; flow rate, 1 mL min^-1^; injection temperature, 280 ^o^C; oven temperature 120 ^o^C (0 min) increasing to 240 ^o^C over a period of 6 min, then increasing to 260 ^o^C at the rate of 6 ^o^C min^-1^, finally increasing to 280 ^o^C at the rate of 40 ^o^C min^-1^ and it was held at this temperature for 5 min. The output from the GC column entered into the ionization chamber of the mass spectrometer via an interface tube maintained at 260 ^o^C. Mass spectrometry (EI, 70 eV, ion source temperature, 230 ^o^C, solvent delay, 3 min) was done in full scan mode. Quadrupole scan monitoring was at m/z 50-550.

**Supplementary table S1. The contents of main fatty acids in WCO**

|  | Palmitic Acid  C16:0 | Stearic Acid  C18:0 | Oleic Acid  C18:1 | Linoleic Acid  C18:2 | Linolenic Acid  C18:3 |
| --- | --- | --- | --- | --- | --- |
|  |
| RT/min | 6.469 | 7.600 | 7.469 | 7.444 | 7.487 |
| wt% | 18.16 | 8.12 | 40.72 | 25.81 | 7.19 |

**2 Characterizations**

**2.1 The GC results of fatty acids in WCO after hydrolysis.**

**
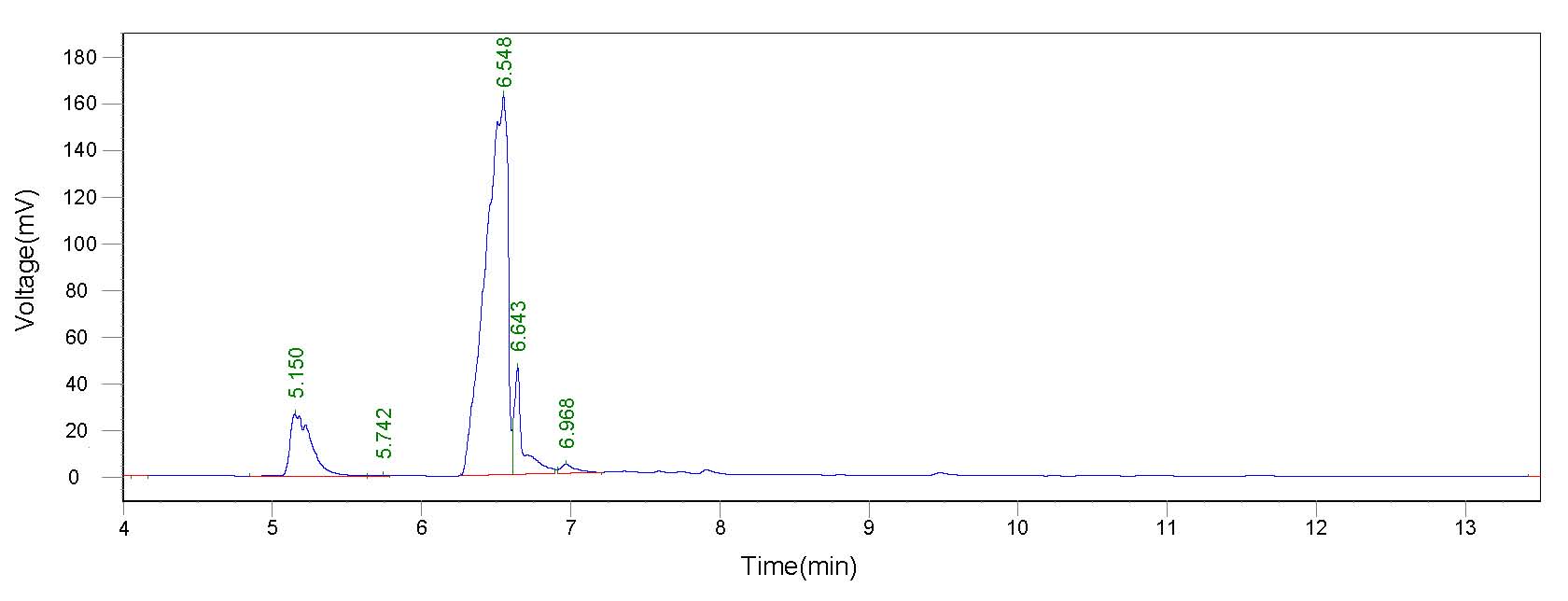
**

C16:0

C18:0

C18:1/2/3

**Figure S2. GC results of fatty acids methyl ester in WCO after hydrolysis.** GC measurements were performed on a GC112A system with flame ionization detector (FID). The carrier gas was nitrogen; flow rate of nitrogen, 11 mL min^-1^; flow rate of hydrogen, 29 mL min^-1^; flow rate of air, 252 mL min^-1^; injection temperature, 290 ^o^C; oven temperature 200 ^o^C (3 min) increasing to 280 ^o^C at the rate of 15 ^o^C min^-1^. It was held at this temperature for 30 min.

**2.2 The GC results of fatty acids in WCO after alkylation.**

**
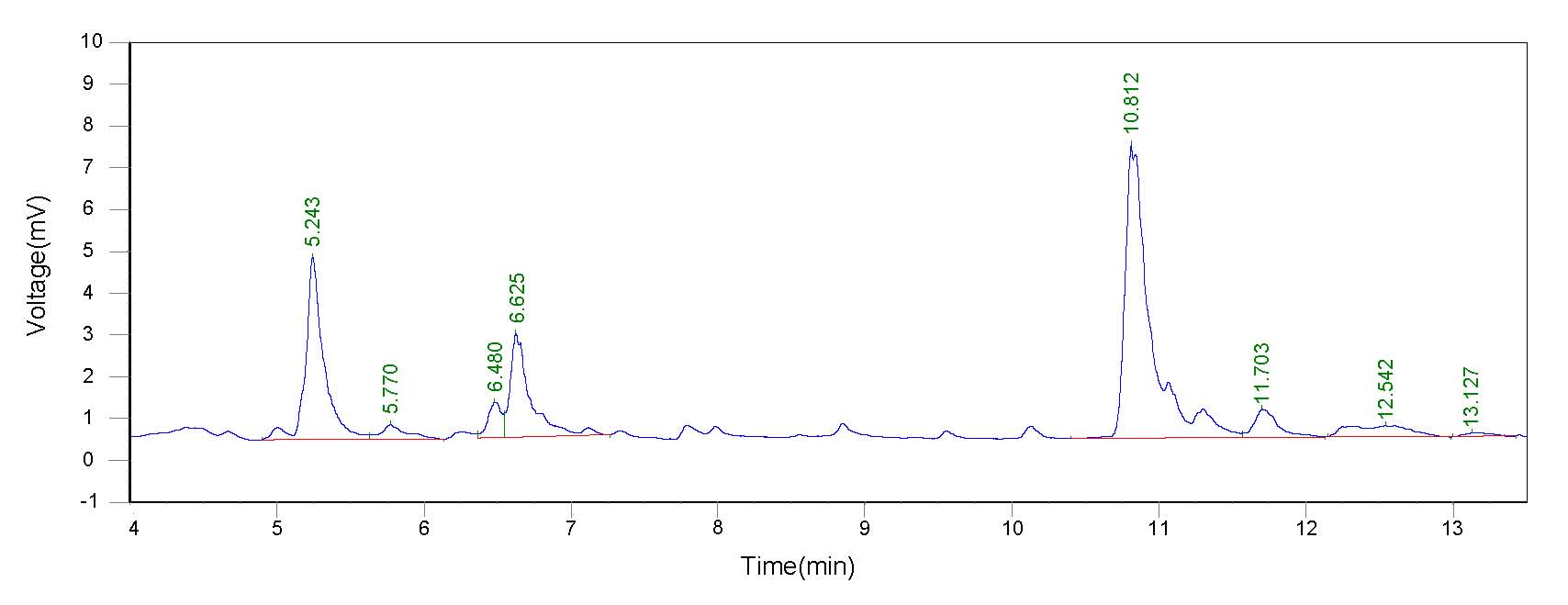
**

C16:0

C18:0

Phenyl fatty acids

**Figure S3. GC results of fatty acids methyl ester in WCO after hydrolysis.** GC measurements were performed on a GC112A system with flame ionization detector (FID). The carrier gas was nitrogen; flow rate of nitrogen, 11 mL min^-1^; flow rate of hydrogen, 29 mL min^-1^; flow rate of air, 252 mL min^-1^; injection temperature, 290 ^o^C; oven temperature 200 ^o^C (3 min) increasing to 280 ^o^C at the rate of 15 ^o^C min^-1^. It was held at this temperature for 30 min.

We adopted Friedel-Crafts alkylation as the first step to modify the double bonds in unsaturated acids. For multi-unsaturated acids (like linoleic acid and linolenic acid), most of them were added with only one benzene from the chromatographic results, although they have more than one double bond initially. The reason for this may be explained by strong steric hindrance nearing the benzene ring. After the first double bond was added with a benzene ring, the probability of another addition of benzene is very low. A conclusion can be drawn from several repeated experiments that most of the products containing only one benzene. Fatty acyl amides play an important role in the preparation process of zwitterionic surfactants as intermediates. They are generally manufactured by the reaction of fatty acids with diamines at high temperature^33-38^. Fatty acyl chloride was introduced as intermediates for synthesis of fatty acid amide in our research, due to its feeble steric hindrance and high reactivity. Hence the subsequent amidation procedure with diamines presented mild reaction condition, less side reaction, low energy consumption and high yield^39^.

**2.3 ESI-HRMS spectrum of PFAPMA.**

**
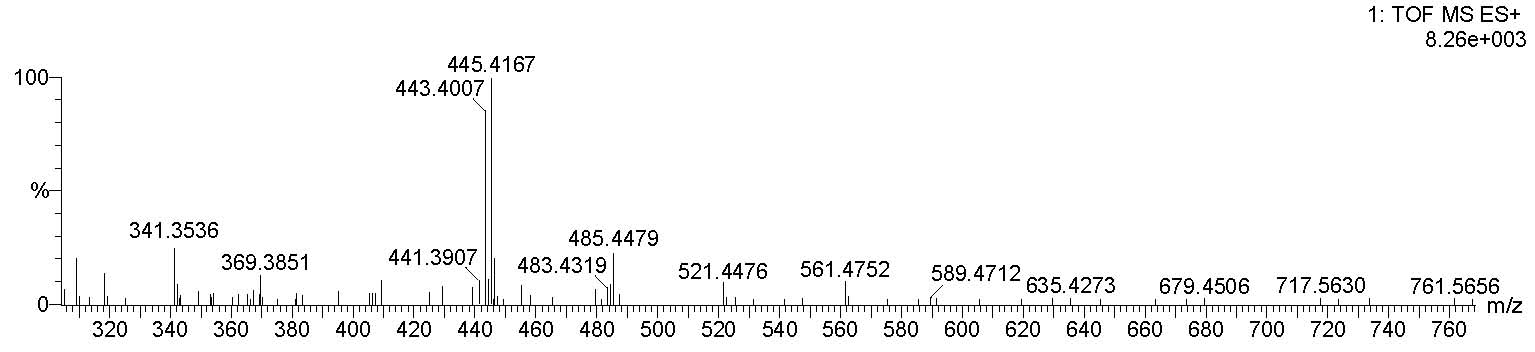
**

**Figure S4. ESI-HRMS spectrum of PFAPMA.**

ESI HRMS: m/z [M+H]^+^ calcd for: 341.3532 (C16:0), 369.3845 (C18:0), 441.3845 (C18:2:pheynl), 443.4001 (C18:1:phenyl), 445.4158 (C18:0:pheyl); found: 341.3536, 369.3851, 441.3907, 443.4007, 445.4167.

**2.4 ESI-HRMS spectrum of PFAPMB.**

**
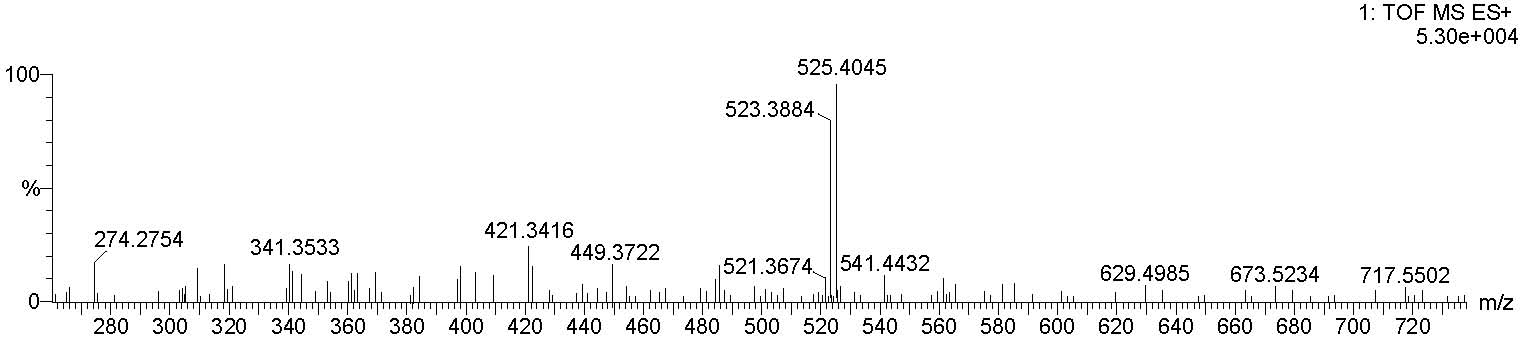
**

**Figure S5. ESI-HRMS spectrum of PFAPMB.**

ESI HRMS: m/z [M+Na]^+^ calcd for: 421.3406 (C16:0), 449.3719 (C18:0), 521.3719 (C18:2:pheynl), 523.3875 (C18:1:phenyl), 525.4134 (C18:0:pheyl); found: 421.3416, 449.3722, 521.3674, 523.3884, 525.4045.

**2.4 ^1^H NMR spectrum of PFAPMB.**

**
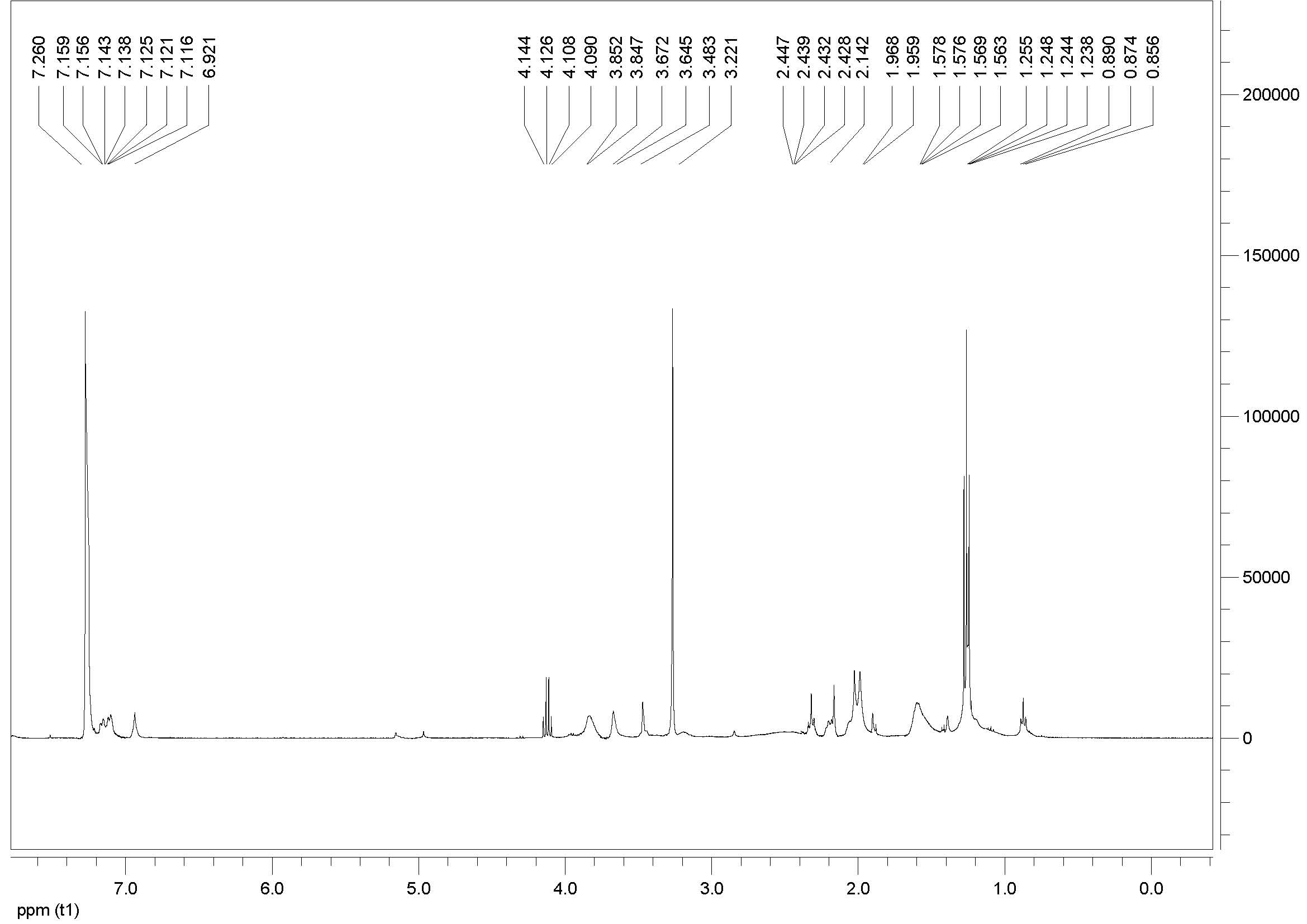
**

l

k

j

i

h

g

f

e

d

c

b

a

m

**Figure S6. ^1^H NMR spectrum of PFAPMB.**

^1^H NMR (400 MHz, CDCl_3_): δ 7.159-7.116 (-C_6_**H_5_**), 6.921 (-CO-N**H**-), 4.144-4.090 (-C**H**=C**H**-), 3.852-3.847 (-NH-C**H_2_**-CH_2_-), 3.672-3.645 (-N(CH_3_)_2_-C**H_2_**-COO), 3.483 (-C**H_2_**-N(CH_3_)_2_-), 3.221 (-N(C**H_3_**)_2_-), 2.447-2.428 (-C**H**-C_6_H_5_), 2.142 (-C**H_2_**-CO-), 1.968-1.959 (-NH-CH_2_-C**H_2_**-CH_2_-N(CH_3_)_2_-), 1.578-1.563 (-C**H_2_**-CH-; -C**H_2_**-CH_2_-CO-), 1.255-1.238 (-(C**H_2_**)_n_-CH_3_), 0.890-0.856 (-CH_2_-C**H_3_**).

Hydrogen atoms can be divided into the following kinds according to their chemical environment.: a) -CH_2_-C**H_3_**, b) -(C**H_2_**)_n_-CH_3_, c) -C**H_2_**-CH-; -C**H_2_**-CH_2_-CO-, d) -NH-CH_2_-C**H_2_**-CH_2_-N(CH_3_)_2_-, e) -C**H_2_**-CO-, f) -C**H**-C_6_H_5_, g) -N(C**H_3_**)_2_-, h) -C**H_2_**-N(CH_3_)_2_-, i) -N(CH_3_)_2_-C**H_2_**-COO, j) -NH-C**H_2_**-CH_2_-, k) -C**H**=C**H**-, l) -CO-N**H**-, m)-C_6_**H_5_**.

**Figure S7. The chemical environment groups of hydrogen atoms in PFAPMB.**

**3 Surface properties**

The inflection point in the plot of SFT versus surfactant concentration is commonly referred to as the critical micelle concentration (CMC) and indicates the point where the air-water surface is saturated with surfactant monomers and, thus, micelles start to form. Critical surface tension (SFT_CMC_) refers to the SFT at CMC. Because the CMC values of bio-based zwitterionic surfactants are low enough and surfactants are purified. So the activity coefficients can be approximated to 1, the concentration of surfactant can be used to replace the activity of surfactant in the usual form of Gibbs equation. Maximum surface excess concentration (Γ_max_) calculated by the following Gibbs equation (1).

$\Gamma_{\max}=\text{-}\frac{1}{2.303\eta RT}(\frac{\partial\text{SFT}}{\partial\lg C})_{T}$ (1)

where *C* is the concentration of surfactant aqueous solution, *R* = 8.314 J/mol/K, *T* = 298.15 K, *SFT* is expressed in mN/m, *η* is a constant and depends on the number of species constituting the surfactant adsorbed at the interface. For zwitterionic surfactants, it is generally assumed that the value of *η* should be set at 1, because no counterion adsorption is expected if the cationic and anionic portions of the molecule are genuinely internally associated. The minimum area occupied per surfactant molecule (A_min_) at air/water interface is related to the surface excess Γ_max_ as follows.

$A_{\min}=(N_{A}\Gamma_{\text{max}})^{-1}\times10^{16}$ (2)

where *N*_A_ is the Avogadro constant.

**4 Contact angle**

The contact angle *θ* of 0.500 g L^-1^ PFAPMB solution was measured using the sessile drop technique on hydrophobic solid substrates, which has an average 92^o^ contact angle on the three phase contact gas/double distilled water/solid. A 2 μL solution drop was introduced onto the solid substrate through a microsyringe. The contact angles were measured by taking photographic images after adding the solution for 60 s. The measurement was repeated 5 times.

**Supplementary table S2. The contact angles of PFAPMB**

| The surfactant | *θ*_1_ | *θ*_2_ | *θ*_3_ | *θ*_4_ | *θ*_5_ | *θ*_average_ |
| --- | --- | --- | --- | --- | --- | --- |
| PFAPMB | 37.67 | 38.87 | 38.69 | 39.23 | 39.48 | 38.79 |

**5 Emulsification property**

The emulsification property of PFAPMB was obtained at room temperature. It was determined by giving ten downward stokes to a mixture of 10 mL 0.500 g L^-1^ PFAPMB solution and 10 mL liquid paraffin in a graduated test tube. The time taken for separation of 3 mL and 5 mL aqueous phase solution was recorded (t/s). The recorded time was repeated for 3 times.

**Supplementary table S3. The time taken for separation of 3 mL and 5 mL aqueous phase solution**

| The surfactant | 3 mL | | | | 5 mL | | | |
| --- | --- | --- | --- | --- | --- | --- | --- | --- |
|  | t_3_-_1_ | t_3_-_2_ | t_3_-_3_ | $\overline{\text{t}_{3}}$ | t_5_-_1_ | t_5_-_2_ | t_5_-_3_ | $\overline{\text{t}_{5}}$ |
| PFAPMB | 152 | 172 | 186 | 170 | 285 | 301 | 320 | 302 |

**6 Foaming property**

The foaming property of bio-based zwitterionic surfactants was obtained according to the Ross-Miles test at 40 ^o^C. The foam heights at 0 min and 10 min were recorded (h/mm)。The recorded height was repeated for 3 times.

**Supplementary table S4.** The foam heights at 0 min and 10 min

| The surfactant | 0 min | | | | 10 min | | | |
| --- | --- | --- | --- | --- | --- | --- | --- | --- |
|  | h_0_-_1_ | h_0_-_2_ | h_0_-_3_ | $\overline{\text{h}_{0}}$ | h_10_-_1_ | h_10_-_2_ | h_10_-_3_ | $\overline{\text{h}_{10}}$ |
| PFAPMB | 74 | 73 | 72 | 73 | 70 | 70 | 69 | 70 |

**Supplementary References**

33 Chu, Z. L. & Feng, Y. J. Vegetable-Derived Long-Chain Surfactants Synthesized via a "Green" Route. *ACS Sustainable Chem. Eng.* **1**, 75-79, (2013).

34 Kumar, R., Kalur, G. C., Ziserman, L., Danino, D. & Raghavan, S. R. Wormlike micelles of a C22-tailed zwitterionic betaine surfactant: From viscoelastic solutions to elastic gels. *Langmuir* **23**, 12849-12856, (2007).

35 Sreenu, M., Nayak, R. R., Prasad, R. B. N. & Sreedhar, B. Synthesis, surface and micellar properties of sodium N-oleoyl amino acids. *Colloid. Surface. A.* **449**, 74-81, (2014).

36 Sreenu, M., Rao, B. V. S. K., Prasad, R. B. N., Sujitha, P. & Chityala, G. K. Synthesis, surface and biological properties of sodium N-acyl isoleucines. *Eur. J. Lipid Sci. Technol.* **116**, 193-206, (2014).

37 Miao, S., Wang, P., Su, Z. & Zhang, S. Vegetable-oil-based polymers as future polymeric biomaterials. *Acta Biomater.* **10**, 1692-1704, (2014).

38 Corma, A., Iborra, S. & Velty, A. Chemical Routes for the Transformation of Biomass into Chemicals. *Chem. Rev.* **107**, 2411-2502, (2007).

39 Zhang, Q. Q., Cai, B. X., Gang, H. Z., Yang, S. Z. & Mu, B. Z. A family of novel bio-based zwitterionic surfactants derived from oleic acid. *RSC Adv.* **4**, 38393-38396, (2014).
